# Age-Dependent Hemoglobin A1c Therapeutic Targets Reduce Diabetic Medication Changes in the Elderly

**DOI:** 10.5334/egems.303

**Published:** 2019-08-26

**Authors:** Thomas A. McCormick, John L. Adams, Eric A. Lee, Nicholas P. Emptage, Darryl E. Palmer-Toy, John P. Martin, Benjamin I. Broder, Michael H. Kanter, Anna C. Davis, Elizabeth A. McGlynn

**Affiliations:** 1 Kaiser Permanente Center for Effectiveness & Safety Research, US; 2 Southern California Permanente Medical Group, US; 3 Kaiser Permanente School of Medicine, US; 4 Kaiser Permanente Research, US

**Keywords:** Diabetes Mellitus, Quality of Care, Chronic Illness, Clinical Practice Pattern, Electronic Health Record, Quality Improvement

## Abstract

**Objective:**

To assess whether implementation of age-dependent therapeutic targets for high hemoglobin A1c (HbA1c) changed clinicians’ ordering of diabetes medications for older adults.

**Background:**

In 2016, Kaiser Permanente Southern California (KPSC) changed the therapeutic targets for alerting clinicians about high HbA1c results in the electronic health record, KP HealthConnect (KPHC). Previously, all HbA1c results ≥7.0 percent were flagged as high in adult patients with diabetes. Starting in 2016, HbA1c therapeutic targets were relaxed to <7.5 percent for patients age 65 to 75, and to <8.0 percent for patients over age 75 to reduce treatment intensity and adverse events.

**Methods:**

This retrospective analysis used logistic regression models to calculate the change in odds of a medication change following an HbA1c result after age-dependent HbA1c flags were introduced.

**Results:**

The odds of medication change decreased among patients whose HbA1c targets were relaxed: Odds Ratio (OR) 0.72 (95 percent CI 0.67–0.76) for patients age 65–75 and HbA1c 7.0 percent–7.5 percent; OR 0.72 (95 percent CI 0.65–0.80) for patients over age 75 and HbA1c 7.0 percent–7.5 percent; and OR 0.67 (95 percent CI 0.61–0.75) for patients over age 75 and HbA1c 7.5 percent–8.0 percent. In the age and HbA1c ranges for which the alerts did not change, the odds of medication change generally increased or stayed the same. There was little evidence of medication de-intensification in any group.

**Conclusions:**

These findings suggest that the change in therapeutic targets was associated with a reduction in medication intensification among older adults with diabetes.

## Introduction

In the past decade, largely in response to the regulations and financial incentives put in place by the 2009 Health Information Technology for Economic and Clinical Health (HITECH) Act, adoption of electronic health record (EHR) technology has increased dramatically in the United States. As of 2015, 77.9 percent of office-based physicians possessed a certified EHR system, while 86.9 percent possessed at least basic health-record functionality [1].

In clinical practice, these systems may be used to implement alerts or reminders that encourage the delivery of best practice and/or guideline-concordant care. Electronic record systems have been used to deliver screening reminders [2,3,4,5,6,7,8], vaccination prompts [3,9,10] and immunization alerts [11,12], to flag medication orders for possible drug interactions [13], and to identify patients in need of care based on algorithms run on the electronic data [14,15]. Although published evaluations of these alerts suggest that they are effective in improving care quality, other research in “real world” clinical practice settings suggest that 49 percent to 96 percent of alerts are overridden by clinicians [16]. This occurs primarily as a result of alert fatigue, as clinical staff often receive large numbers of alerts, and may dismiss many of them as a time-management strategy [17]. The mechanism by which alert fatigue leads to alert overrides is not well-understood: alerts may be overridden because they interrupt clinicians’ workflows (i.e., they must be routinely bypassed to access more pertinent sections of the patient’s medical record), but multiple (and possibly competing) alerts encountered frequently may lead to cognitive overload, in which considerable effort is required to identify the most important alerts. Another possible mechanism involves desensitization, in which repeated exposure to the same alert causes it to be dismissed more frequently over time. At the request of clinical leaders, we took advantage of a natural experiment in Kaiser Permanente Southern California (KPSC) to examine whether changes to which hemoglobin A1c (HbA1c) results were flagged as abnormal were associated with differences in subsequent physician medication orders.

Glycemic control is a hallmark feature of effective diabetes care. For non-pregnant adults, the general blood glucose target level is an HbA1c of <7.0 percent [18]. However, older adults have been excluded from most of the large trials that have established data on glucose control targets, and as a result, there is less certainty about the ideal target HbA1c level in this age group [19]. Evidence from the Action to Control Cardiovascular Risk in Diabetes [ACCORD] trial [20] and others has suggested that intensive glucose management in older adults can be harmful [19]. However, for some groups of older adults, intensive glucose control may be appropriate; the risks and benefits of tight glucose control in older adults are dependent on factors like frailty, life expectancy, duration of diabetes, and comorbidities [19].

Overarching goals set by the American Diabetes Association (ADA) are for an HbA1c <7.5 percent in healthy older adults, <8.0 percent in older adults with complex or intermediate health status, and <8.5 percent in those with very complex or poor health [21,22]. The American College of Physicians sets a general HbA1c goal between 7.0 percent and 8.0 percent in most patients [23]. Both organizations recommend that providers use an individualized approach to setting treatment targets.

In KPSC, clinicians receive laboratory results through an Epic-based electronic health record system (Epic Systems, Verona, WI), known internally as KP HealthConnect^®^ (KPHC). This system is used for documentation and coordination of care provided by over 7,000 physicians as well as the other clinical and support staff throughout its 15 hospitals and 233 medical centers. At the time that a new HbA1c result is available, the laboratory system sends a message in KPHC to the patient’s Primary Care Provider (PCP). If the HbA1c is above the threshold to be considered abnormally high, this message includes an alert consisting of a yellow highlight over the HbA1c value. There is also text indicating that a less stringent goal may be appropriate for an individual patient with a history of severe hypoglycemia, advanced microvascular complications, or extensive comorbid conditions. The flags indicating high HbA1c are part of the physician’s laboratory result inbox and do not require immediate attention from a clinician in order to complete documentation or other work.

Historically, all results for patients with diabetes with HbA1c ≥7.0 percent were flagged as high. In January of 2016, the laboratory system implemented a change in the range of HbA1c values that were flagged as high. Starting in 2016, HbA1c therapeutic targets were relaxed to <7.5 percent for patients age 65 to 75, and to <8.0 percent for patients age 76 and above [24]. This change was based on the hypothesis that more stringent therapeutic targets may result in overly intensive management of glucose in older diabetic patients. We address the question: are age-dependent therapeutic HbA1c targets associated with less intensive glucose management among persons age 65 and older who have diabetes, compared to targets that do not differentiate based on age?

By relaxing the HbA1c targets that triggered these alerts for older adults, KPHC brought the alert thresholds in line with the general guidelines for glucose control in patients over age 65. Clinicians continued to make individualized assessments of the appropriate HbA1c target, and managed medications accordingly, but were no longer prompted by the medical record system to act on HbA1c levels that were within the general goals set by the ADA.

## Methods

### Data sources

We used secondary data drawn from existing data systems. We assembled clinical and demographic data for the membership of KPSC. The membership of KPSC is diverse and is generally representative of the greater population of Southern California [25]. Data sources included Kaiser Permanente membership records, integrated claims, electronic medical records, and administrative data repositories.

This analysis was undertaken at the request of clinical leaders to assess whether changing the alerting logic for high HbA1c values in KPHC would affect clinician behavior in diabetes management. It was determined to be a quality improvement project, and was exempt from IRB review.

### Population Definition

This retrospective analysis of HbA1c results included the time from January 1, 2015 through December 31, 2016, to capture 12 months before and after the change in the flags for high HbA1c laboratory values. However, to identify patients with diabetes, we used an extended look-back period and applied the Surveillance, Prevention, and Management of Diabetes Mellitus (SUPREME-DM) algorithm [26] to data between 2008–2016. We excluded patients with type 1 diabetes from the analysis, using the first two criteria of Klompas’ optimized algorithm [27].

After applying the SUPREME-DM and Klompas algorithms, we limited the study population to patients age 55 and older who had at least one HbA1c result in 2015 or 2016, excluding the last six weeks of each year (to allow a full six weeks to measure outcomes).

We extracted membership data for all patients. Patients with membership gaps during 2014–2016 between the first month after the initial indication of diabetes (per the SUPREME-DM algorithm) and the last month of membership were excluded. We also excluded those who were enrolled for six or fewer months of membership in 2015–2016 after the first indication of diabetes.

Patients with bariatric surgery procedures or any encounters in a skilled nursing facility (SNF) during the analysis period were excluded. Although we did not expect that any women in the analysis population would be pregnant (because of the age ranges of interest), patients with any indication of pregnancy were excluded on the assumption that the patient’s birth date was in error (there were 141 such patients).

Medication orders were used as a proxy for clinician action following high HbA1c results. Orders were used rather than dispenses so that the analysis was not complicated by patient adherence and because the intent was to examine physician behavior rather than patient behavior. To construct the medication list, all National Drug Codes (NDCs) in the Healthcare Effectiveness Data and Information Set (HEDIS) 2015 and 2016 lists, augmented by a list developed by colleagues [28] were combined. We used these NDCs and the generic drugs associated with these medications to find diabetes medication orders in KPHC.

We categorized the medications based on the ten categories in the HEDIS 2015 specification: metformin, sulfonylurea, insulin, glucagon-like peptide-1 agonist (GLP1), dipeptidyl peptidase-4 inhibitor (DPP4), sodium-glucose cotransporter 2 inhibitor (SGLT2), amylin analog, alpha-glucosidase inhibitor, meglitinide, and thiazolidinedione. Medications present as of the date of the HbA1c result were determined based on the ordering date and the end of the orders. Missing end date values were set to the end of the analysis period, and the end of an order was assigned based on the earlier of the end date and the discontinue date (if it existed). On a given date, the combination of open orders was used to determine the combination of treatments that the patient was intended to be using. The patient’s treatment was calculated as of the day after the order, to account for the fact that orders for different medications can start and end on the same day.

Medication dose was calculated for oral medications because the source data did not include dose information. A text-parsing algorithm was developed to calculate the number of tablets per day from the Sig field associated with the order. The number of tablets per day was capped at 8, and tablets per day were multiplied by strength in milligrams to calculate daily dose. Dispensing data were used to validate the text-parsing algorithm. If dispense data for the same medication were available shortly after the order, and if the dose calculated from a dispense of the same drug was different from the calculation based on Sig, we used the dose based on dispense in the analysis. If multiple dispenses or multiple orders happened on the same day, the event with the later end date was used for the dose calculation. Patients were censored after the first insulin order because an accurate daily dose could not be calculated for most types of insulin.

### Outcomes

The outcome we examined was a change in medication management. To detect a change in the odds of medication management, we scanned the 6 weeks following each HbA1c result for the first date with any medication orders. If such a date existed, an intensification or de-intensification of medication or dose was considered a change in medication management. Combinations of orders that were not a clear intensification or de-intensification (for example, intensification of one medication and de-intensification of another) were grouped with the “no change” outcome.

### Statistical Analysis

We used logistic regression to compare the odds of a change in medication before and after the implementation of age-dependent therapeutic targets that raised the HbA1c threshold for alerting clinicians to high results in older adults. We constructed regression models to compare the odds of having a medication change for patients ages 65 to 75, and 76 and up in 2016 versus 2015, as well as for patients ages 55 to 64 (for whom there were no changes to the alert algorithm in 2016). The covariates in the models include gender, race/ethnicity, and age (in years) within each age group. A separate model was fit for each age group.

The main model to compare the odds of medication change in 2016 with the odds in 2015 was a binary outcome model for any medication change (intensification or de-intensification). Standard errors were calculated with generalized estimating equations, to account for the correlations associated with a patient contributing multiple HbA1c results to the analysis.

Several sensitivity analyses were conducted, including:

- Alternative binary models of intensification vs. “not intensification” (i.e., no change or de-intensification), and de-intensification vs. “not de-intensification” (i.e., no change or intensification).
- Three-level ordinal multinomial regression, in which intensification, no change, and de-intensification were considered separate outcomes.
- Adding the individual Charlson comorbidities as covariates in the model, based on International Classification of Diseases, Ninth Revision (ICD-9) and ICD-10 diagnosis codes [29].
- Adding HbA1c results from the last six weeks of each calendar year.
- Excluding results from January of each year, because the age-dependent therapeutic targets were implemented over the course of January 2016.
- Excluding the approximately 2 percent of HbA1c results that were ordered in an inpatient setting.
- Grouping combinations of orders that were not a clear intensification or de-intensification with the “change” outcome rather than the “no change” outcome.

The analysis was conducted with SAS Enterprise Guide 7.1 (SAS Institute, Cary, NC).

## Results

**Figure 1:**
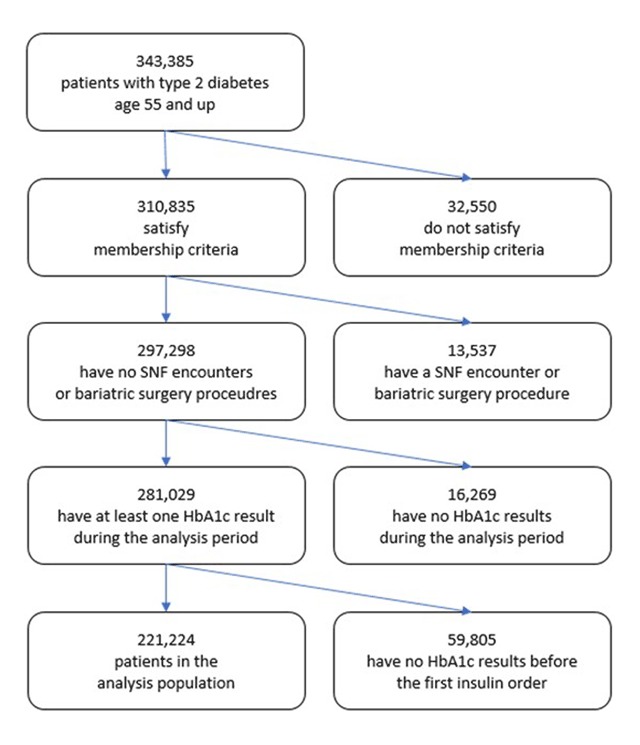
CONSORT diagram with detailed patient exclusion counts.

We identified 343,385 patients with type 2 diabetes age 55 and older in KPSC. Of these, 221,224 satisfied all criteria to be included in the analysis. The detailed counts of patients included and excluded can be seen in Figure 1.

### Patient Demographics

Patient demographics and characteristics of HbA1c results in the groups are outlined in Table 1. Note that the patient counts in the age groups do not sum to the count of all unique patients because approximately 6 percent of patients contributed to multiple age groups during the analysis (that is, they moved from age 64 to 65 or from age 75 to 76 during 2015 or 2016). Over 50,000 HbA1c results in 2016 were below the age-dependent cutoff values and thus did not generate an alert to the clinician (but would have based on the 2015 cutoff values).

**Table 1:** Analysis population demographics.

	All Patients	Patients Contributing to Each Age Group		

		55–64	65–75	76 and up

Patient count	221,224	91,510	94,369	49,569
% Female	49	48	50	52
Mean age at end of analysis (SD)	69.1 (9.0)	60.7 (3.2)	70.5 (3.4)	82.3 (4.8)
HbA1c result count	711,400	266,617	291,024	153,759
Mean HbA1c value (%) (SD)	7.2 (1.2)	7.4 (1.3)	7.1 (1.1)	6.9 (1.0)
Race/Ethnicity				
% Non-Hispanic Asian/Pacific Islander	16	16	17	14
% Non-Hispanic Black	12	12	12	13
% Hispanic	32	37	30	26
% White	36	30	37	44
% Other	4	5	4	2
Mean number of HbA1c results (SD)				
in full analysis period	3.2 (1.6)	2.9 (1.6)	3.1 (1.5)	3.1 (1.5)
in 2015 (pre-period)	1.6 (1.0)	1.4 (1.1)	1.5 (1.0)	1.5 (1.0)
in 2016 (post-period)	1.6 (1.0)	1.5 (1.1)	1.6 (1.0)	1.6 (1.0)

### Medication Changes Following HbA1c Results

The 221,224 members included in our study had 711,400 HbA1c results that were included in the analysis. Approximately half of HbA1c results were below 7.0 percent. The 55 to 64 and 65 to 75 age groups had similar counts of HbA1c results, and the 76 and up age group had approximately half as many as these two groups. The count of HbA1c results by age group and HbA1c range is displayed in Appendix 1.

**Figure 2:**
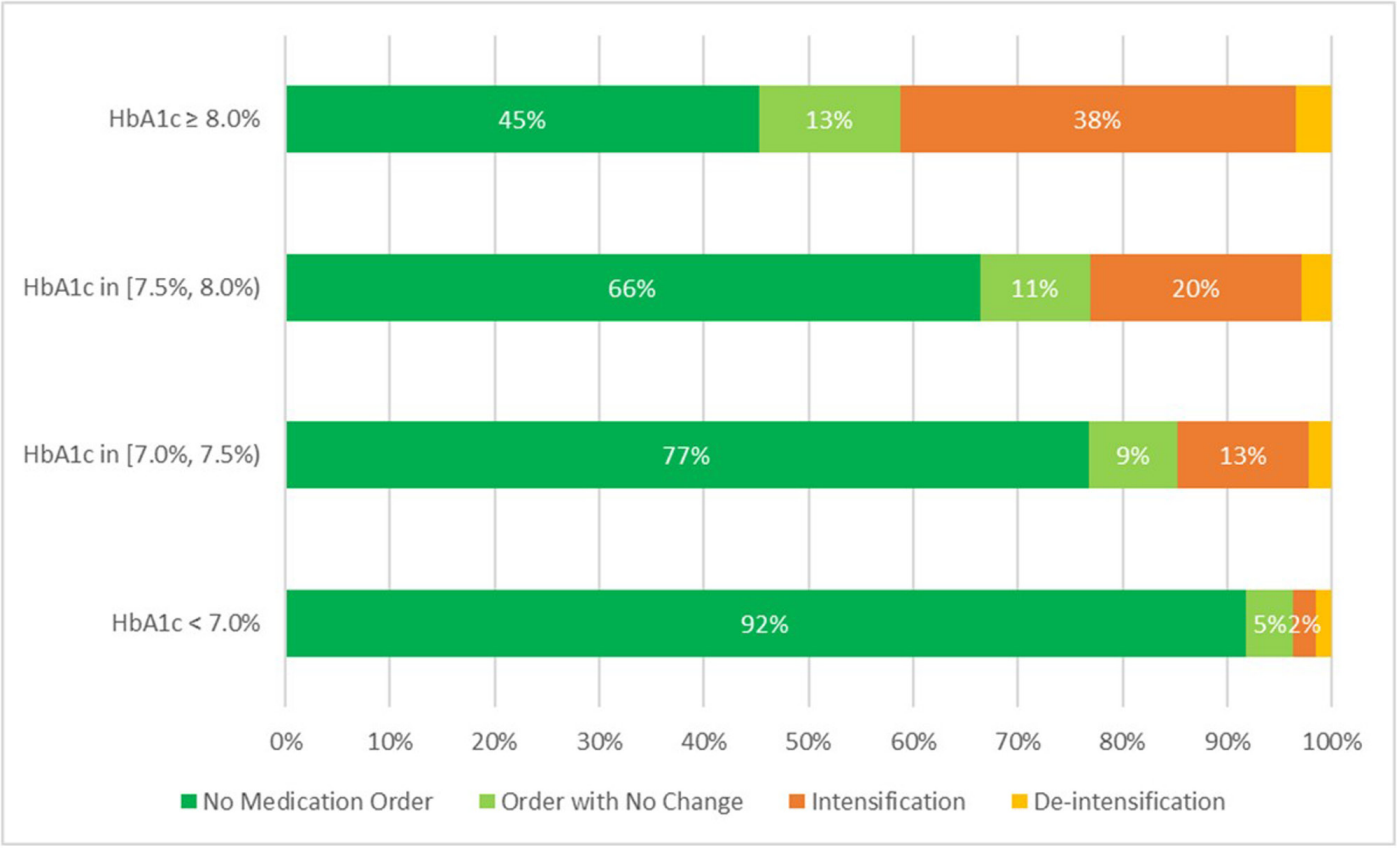
Fraction of HbA1c results with an order in the following 6 weeks.

Figure 2 summarizes the fraction of HbA1c results with a medication order in the 6 weeks after the laboratory test. For the majority of HbA1c results overall (78 percent), and for all levels of HbA1c results except the highest level (HbA1c greater than or equal to 8.0 percent) there was no medication order in the subsequent 6 weeks. However, the fraction of results that are followed by an order increases as the HbA1c value increases, and the fraction with a change that represents an intensification in medications or dose also increases as the HbA1c value increases. In these simple univariate statistics, there are few orders representing a de-intensification in medications or in dose, but among those with medication orders, the fraction with medication deintensification rises as HbA1c drops. Appendix 2 shows the data in Figure 2 separately for each age group.

### Modeling the Odds of a Medication Change

**Figure 3:**
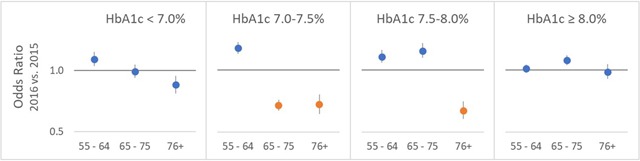
Odds ratios (and 95% CIs) for odds of a change in medication in 2016 vs. 2015, distinguishing HbA1c targets that did change (orange) and did not change (blue) to age-dependent values in 2016.

The results from the models of medication change are listed in Table 2 and displayed in Figure 3. An odds ratio below 1.0 (solid grey line) indicates lower odds of a medication change in 2016 compared to 2015. The four panels in the plot show the odds ratios in the four HbA1c ranges of interest, with a point estimate and 95 percent confidence interval from each age group model. The three HbA1c-age combinations for which the flags changed to reflect age-dependent cutoffs in 2016 are highlighted in orange.

**Table 2:** Odds ratios and 95% CI’s for odds of a change in medication in 2016 vs. 2015.

HbA1c Range	Age Group	Odds Ratio	Low 95% CI	High 95% CI

HbA1c < 7.0%	55–64	1.09	1.04	1.15
HbA1c < 7.0%	65–75	0.99	0.94	1.05
HbA1c < 7.0%	76+	0.88	0.81	0.96
HbA1c 7.0%–7.5%	55–64	1.18	1.13	1.23
HbA1c 7.0%–7.5%	65–75	0.72	0.67	0.76
HbA1c 7.0%–7.5%	76+	0.72	0.65	0.80
HbA1c 7.5%–8.0%	55–64	1.11	1.06	1.16
HbA1c 7.5%–8.0%	65–75	1.16	1.10	1.22
HbA1c 7.5%–8.0%	76+	0.67	0.61	0.75
HbA1c ≥ 8.0%	55–64	1.01	0.98	1.05
HbA1c ≥ 8.0%	65–75	1.08	1.04	1.12
HbA1c ≥ 8.0%	76+	0.99	0.93	1.05

The results are consistent with the hypothesis that the change in the flags is associated with a change in clinician behavior. For the age and HbA1c range combinations in which the flags changed to age-dependent targets in 2016, the odds ratios are approximately 0.7 reflecting lower likelihood of a medication order following the HbA1c test result. For the other ranges, in which the flags stayed the same in 2016, nearly all the odds ratios have confidence intervals that include 1.0 or that are entirely above 1.0, which suggests that a change in medications following an HbA1c result was more likely in 2016 than in 2015.

The exception was the odds ratio for HbA1c results below 7.0 percent in the 76 and up age group, which was less than 1 (OR 0.88, 95 percent CI 0.81–0.96). We modeled the results separately for patients who were already using medications and those who were not because those who were not on medications could not have their treatment de-intensified. It appears that the low odds ratio may be due more to the group of patients not already using medications, suggesting that older patients are less likely to start their first medications following low HbA1c results in 2016. The odds ratios for these two sets of models are displayed in Appendix 3.

None of the results were sensitive to the analyses described in the Statistical Analysis section. In particular, there was no evidence of a change in de-intensification from the binary or the three level models, which was probably due to the small number of cases of de-intensification in the data. Thus, it appears that the differences in the odds of medication change in 2016 are associated mainly with a decrease in the odds of intensification of medication or dose.

## Discussion

In this analysis, we examined a natural experiment related to a change in flags that identify high HbA1c results within the laboratory results module of a comprehensive electronic medical records system, KPHC, for older patients with type 2 diabetes. We compared the time before and after the change in flags to understand the association between the change in flags and the odds of a medication change following HbA1c results. We found an association between the age-dependent flags and the odds of a medication change. Specifically, we found evidence of lower odds of intensification of medications in the age groups and HbA1c ranges in which the therapeutic targets were relaxed. This suggests that clinicians may indeed make fewer changes to medications when they do not receive alerts about HbA1c results that are above 7.0 percent but may be within acceptable bounds based on patient age, according to current consensus guidelines.

Our findings are consistent with the literature on the potential of EHR based alerts/flags to encourage changes in physician prescribing behavior with the important caveat that “alert fatigue” may reduce the effectiveness of these methods. The strength of this study is the ability to examine the real-world effectiveness of a strategy to reduce unnecessary alerting of clinicians to age-appropriate HbA1c values in the diabetes population in a large integrated care system. Our relatively large sample size combined with pre-post comparisons, different target ranges by age, detailed clinical data, and the absence of a medication management change in the age group where flags were unchanged make a compelling case for the effectiveness of the flags. Clinical leaders were encouraged to learn that the change in flags appeared to affect clinician behavior, and discussed recommending that this change in the KPHC HbA1c alerting algorithm be implemented across all Kaiser Permanente regions.

There are several limitations in this study. First, there is the possibility that secular changes in practice patterns were leading to less treatment intensification in these age groups even without the implementation of the age dependent flags. Individual clinicians may review updated society guidelines and other materials that could have exposed them to the current ADA recommendations. Within KPSC, the chiefs of the General Internal Medicine, Family Medicine, and Endocrinology departments were notified of the proposed change in therapeutic targets and asked to provide feedback; it is unknown whether they cascaded the information to other providers. All physicians received an email notification about the change shortly before it went into effect. To our knowledge, there were no concurrent changes in clinical practice guidelines or additional communications about changes in diabetes care to physicians or other supporting personnel. Although some of what we see in the patterns over time may be attributable to secular trends we cannot estimate, the sharp differences in the age bands and the lack of trend in the under 65 age group suggests that much of the effect may be attributed to the changing flags. This study establishes a hypothesized effect size for future prospective studies which could be undertaken to test causality (with study designs such as randomizing the HbA1c results that received the updated alerts, or a stepped-wedge rollout). The generalizability of these results to other care models must be considered cautiously. The alerts did not rely on any unique features of the staff model although they benefit from the mature implementation of the electronic health record system and the use of population health management analytics for diabetes and many other conditions. The alerts were implemented as they likely would be implemented in any mature EHR system with supporting analytics. Competing physician attention and different incentives in other systems could alter the effectiveness of the alerts. We also were not able to explore the effect of changing the flags on the rate of hypoglycemia events because these events were quite rare in the pre-period as well as the post-period. Ultimately, an understanding of the effects of the flag changes on hypoglycemic events would be useful. It is possible that such analyses could be done once additional years of data with the new alert system in place are available.

## Conclusion

As health systems continue to explore opportunities to use analytics powered by rich clinical data in electronic health records, it is encouraging that changes in a simple and straightforward alert system can have the desired effect on clinician behavior. Most of the research to date has focused on efforts to increase the use of appropriate health care interventions but there is increased interest in effective mechanisms to de-intensify treatment. This work provides an example of one potential successful strategy.

## Additional Files

The additional files for this article can be found as follows:

10.5334/egems.303.s1 Appendix 1. HbA1c result counts by age group and HbA1c range.

10.5334/egems.303.s2 Appendix 2. Fraction of HbA1c results with an order in the following 6 weeks, by age group.

10.5334/egems.303.s3 Appendix 3. Odds ratios from separate models for patients already using and not already using medications as of the HbA1c date.
