# Supplementary material for: Age-Dependent Hemoglobin A1c Therapeutic Targets Reduce Diabetic Medication Changes in the Elderly
Source: EGEMS (Wash DC). 2019 Aug 26;7(1):46. doi: 10.5334/egems.303 (PMC6715934; doi:10.5334/egems.303)
Supplement: Appendix 1. — HbA1c result counts by age group and HbA1c range. [file egems-7-1-303-s1.pdf]

Appendix 1. HbA1c result counts by age group and HbA1c range.

	Age Group			
HbA1c (%)	55 - 64	65 - 75	76 and up	Total
< 7.0	125,512	154,084	93,479	373,075
7.0 - 7.5	51,865	54,346	25,401	131,612
7.5 - 8.0	31,988	35,620	15,696	83,304
≥ 8.0	57,252	46,974	19,183	123,409
Total	266,617	291,024	153,759	711,400
