# Supplementary figures and images for: Age-Dependent Hemoglobin A1c Therapeutic Targets Reduce Diabetic Medication Changes in the Elderly
Source: EGEMS (Wash DC). 2019 Aug 26;7(1):46. doi: 10.5334/egems.303 (PMC6715934; doi:10.5334/egems.303)

## Appendix 2. Fraction of HbA1c results with an order in the following 6 weeks, by age group.

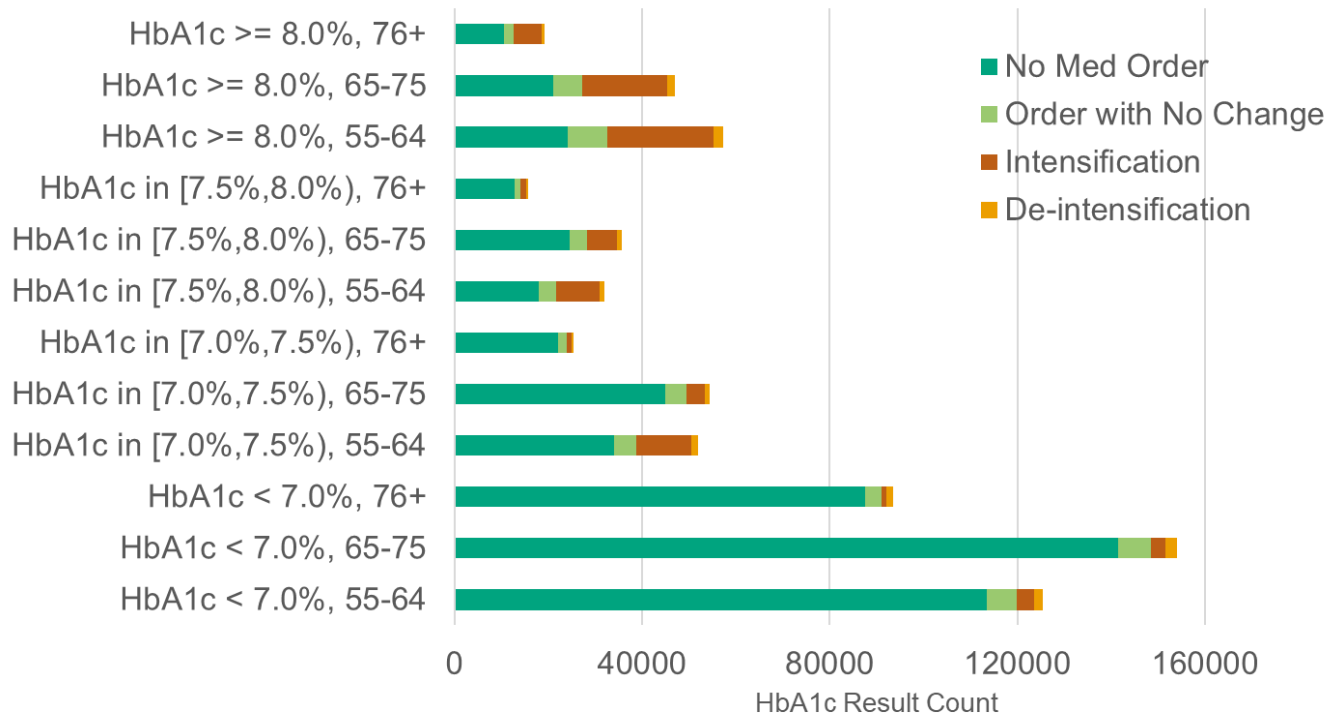

Supplement: Appendix 2. — Fraction of HbA1c results with an order in the following 6 weeks, by age group. [file egems-7-1-303-s2.pdf]
