# Supplementary material for: Age-Dependent Hemoglobin A1c Therapeutic Targets Reduce Diabetic Medication Changes in the Elderly
Source: EGEMS (Wash DC). 2019 Aug 26;7(1):46. doi: 10.5334/egems.303 (PMC6715934; doi:10.5334/egems.303)

Appendix 3. Odds ratios from separate models for patients already using and not already using medications as of the HbA1c date.

Odds ratios (and 95% CIs) for odds of a change in medication in 2016 vs. 2015, distinguishing HbA1c targets that did change and did not change to age-dependent values in 2016, separately for patients already using medications or not using medications as of the HbA1c result date.

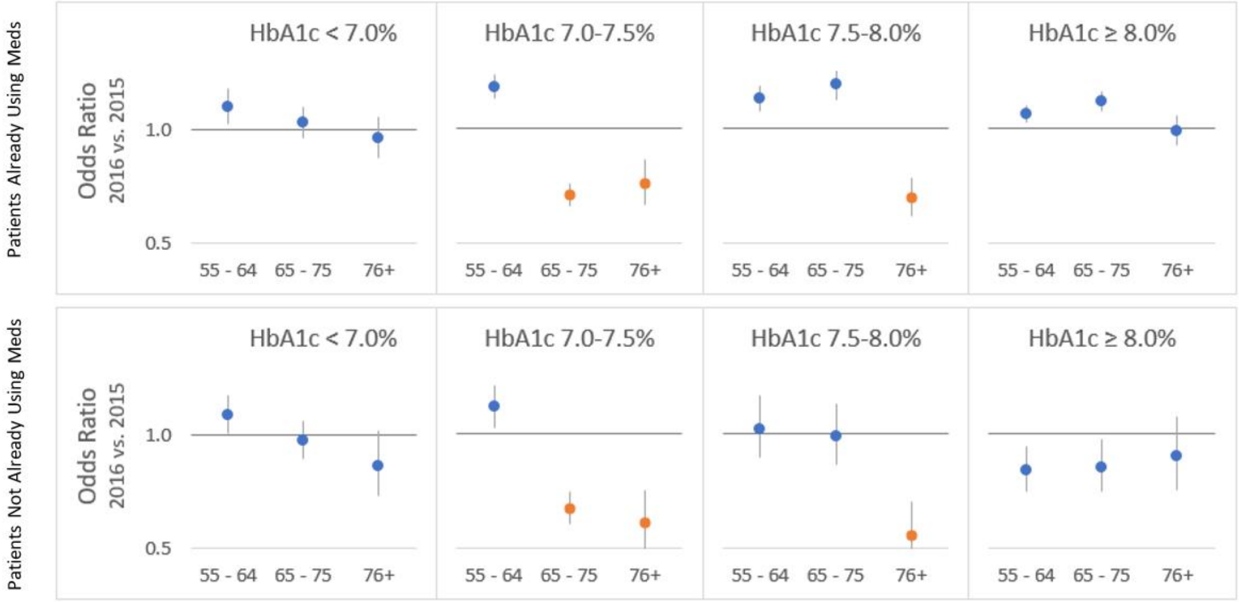

Supplement: Appendix 3. — Odds ratios from separate models for patients already using and not already using medications as of the HbA1c date. [file egems-7-1-303-s3.pdf]
